# Dynamics and Insights into the Unique Ecological Guild of Fungi in Bacteria-Bioaugmented Anaerobic Digesters

**DOI:** 10.3390/jof11010056

**Published:** 2025-01-13

**Authors:** Linda U. Obi, Ashira Roopnarain, Memory Tekere, Jun Zhou, Heng Li, Yuanpeng Wang, Yanlong Zhang, Rasheed A. Adeleke

**Affiliations:** 1 Microbiology and Environmental Biotechnology Research Group, Institute for Soil, Climate and Water, Agricultural Research Council, Arcadia, Pretoria 0083, South Africa; obilindauloma@gmail.com (L.U.O.); roopnaraina@arc.agric.za (A.R.); 2 Unit for Environment Science and Management, North-West University (Potchefstroom Campus), Private Bag X1290, Potchefstroom 2520, South Africa; 3 Department of Environmental Sciences, College of Agriculture and Environmental Sciences, University of South Africa (UNISA), P.O. Box 392, Florida 1710, South Africa; tekerm@unisa.ac.za; 4 Bioenergy Research Institute, College of Biotechnology and Pharmaceutical Engineering, Nanjing Tech University, Nanjing 211816, China; zhoujun@njtech.edu.cn; 5 Key Laboratory of Estuarine Ecological Security and Environmental Health, Tan Kah Kee College, Xiamen University, Zhangzhou 363105, China; 6 Key Laboratory for Chemical Biology of Fujian Province, Department of Chemical and Biochemical Engineering, College of Chemistry and Chemical Engineering, Xiamen University, Xiamen 361005, China; wypp@xmu.edu.cn; 7 Key Laboratory of the Ministry of Education for Coastal and Wetland Ecosystem, College of the Environment & Ecology, Xiamen University, Xiamen 361102, China; ylzhang@xmu.edu.cn; 8 Fujian Key Laboratory of Coastal Pollution Prevention and Control (CPPC), College of Environment & Ecology, Xiamen University, Xiamen 361102, China; 9 Fujian Institute for Sustainable Oceans, Xiamen University, Xiamen 361102, China

**Keywords:** fungal microbiome, anaerobic fungi, biogas production, high-throughput sequencing

## Abstract

Anaerobic digesters host a variety of microorganisms, and they work together to produce biogas. While bacterial and archaeal communities have been well explored using molecular techniques, fungal community structures remain relatively understudied. The present study aims to investigate the dynamics and potential ecological functions of the predominant fungi in bacteria-bioaugmented anaerobic digesters. Eight different anaerobic digesters that contained chopped water hyacinth and cow dung as feedstock at 2% total solids were respectively inoculated with eight different bacterial strains and digested anaerobically in controlled conditions. The diversity and dynamics of the fungal community of the digesters before and after digestion were monitored using high-throughput sequencing of the fungal *ITS2* sub-region of the ribosomal gene. The functional potential of the fungal community was predicted using ecological guild analysis. The dominant fungal phyla were (with relative abundance ≥1%) Ascomycota and Neocallimastigomycota. Ascomycota exhibited over 90% dominance in all treatments after anaerobic digestion (AD). *Aspergillus* sp. was consistently dominant across treatments during AD, while prominent anaerobic fungal genera *Anaeromyces*, *Cyllamyces*, and *Caeomyces* decreased. Ecological guild analysis at genus level showed that the majority of the identified fungi were saprophytes, and diversity indices indicated decreased richness and diversity after AD, suggesting a negative impact of AD on fungal communities in the anaerobic digesters. The multivariate structure of the fungal communities showed clustering of treatments with similar fungal taxa. The findings from this study provide insights into the fungal ecological guild of different bacteria-bioaugmented anaerobic digesters, highlighting their potentials in bacteria-augmented systems. Identification of an anaerobic fungal group within the phylum Ascomycota, beyond the well-known fungal phylum Neocallimastigomycota, offers a new perspective in optimizing the AD processes in specialized ecosystems.

## 1. Introduction

Anaerobic digestion (AD) is an important process in waste treatment and renewable energy production, providing an environmentally sustainable method for converting organic matter into biogas in an anaerobic digester. In recent times, the extensive application of biogas as a source of renewable energy has risen [1]. The production of biogas in anaerobic digesters through the metabolic process of AD involves the complex synergistic interactions of different microbes including fungi, bacteria, and archaea [2]. Fungi have been observed to be vital microorganisms due to their contribution to the breakdown of organic matter. These microorganisms initiate the degradation process by opening up the cells of complex lignocellulosic substrates for subsequent degradation by bacteria and archaea, thereby accelerating the metabolic process [3,4]. Fungi may not be as prevalent as bacteria and archaea in anaerobic digesters due to environmental conditions, and there are limited studies on fungal communities of anaerobic digesters [2]. However, they demonstrate some metabolic activities, particularly during the early stage of AD, also known as the hydrolytic phase. The hydrolytic phase of AD has been identified as the rate-limiting phase in the AD of lignocellulosic substrates and it involves the metabolic activities of fungal organisms, including the production of extracellular enzymes for the potential breakdown of complex lignocellulosic substrates [5,6]. Inhibition of the hydrolytic step could impede the AD process or result in generating recalcitrant intermediates [7]. To optimize the AD process by increasing microbial activity, the incorporation of active microbial strains into the native microbial community of anaerobic digesters (bioaugmentation) is imperative, to enhance the breakdown of biomass [8]. Bioaugmentation has been implicated in enhancing digester performance and mitigating ammonia and salinity stresses, subsequently improving methane production [9].

Some fungi can initiate cellulose degradation by hydrolyzing the complex crystalline structure of lignocellulose, breaking the core β-1,4-glucan bonds through random depolymerization [10]. These fungi are distributed across different fungal phylogenies and most belong to phyla such as Basidiomycota and Ascomycota, as well as the famous anaerobic fungal phylum Neocallimastigomycota [3]. Bacteria are equally beneficial and predominant in biodigesters in terms of biomass degradation. They are also essential at the initial stages of AD, like hydrolysis, acidogenesis, and acetogenesis, while archaea, which are primarily methanogens, utilize substrates including molecular hydrogen to generate methane in the final process of AD, known as methanogenesis [11,12]. Bacterial and archaeal communities of anaerobic digesters have been widely studied using different molecular techniques that are either focused on the *16S rRNA* gene or focused on the metagenome [2,13,14,15,16]. However, only a few studies have reported on the community structure of fungi in bacteria-bioaugmented anaerobic digesters [2,17].

The bioaugmentation of anaerobic digesters with bacteria is ideal due to the resilient and adaptive features of bacteria developed in response to extreme environments. Understanding the metabolic feature of the indigenous and introduced microbial entities of anaerobic digesters is imperative for an efficient AD process. Bioaugmentation with bacteria has been reported to cause a shift in the microbial community of anaerobic digesters, though some studies have reported otherwise [18]. Introducing microbial species with specialized enzymatic capabilities can address key limitations in the anaerobic digestion (AD) of lignocellulosic substrates by enhancing the hydrolysis step, often considered a rate-limiting phase in the process. In this study, the bacterial strains used for bioaugmentation were selected based on their facultative anaerobic characteristics, which allow them to adapt effectively to the digester environment. These strains also demonstrate hydrolytic and cellulolytic abilities when cultivated on sterile carboxymethyl cellulose (CMC) agar, as reported in previous studies [19,20]. *Pseudomonas stutzeri* has shown significant cellulolytic and hydrolytic potential, attributed to the presence of the A1501 cellulase gene [21]. Additionally, *Exiguobacterium* species have exhibited the ability to produce endoglucanase, a form of cellulase, when grown on lignocellulosic substrates such as watermelon peels [22]. *Bacillus cereus* has demonstrated the potential to metabolize lignocellulosic substrates into short-chain fatty acids, a key intermediate in the anaerobic digestion (AD) process [23]. Other cellulolytic bacteria employed in this study include *Lysinibacillus fusiformis*, known for its cellulolytic activity and ability to degrade complex polysaccharides effectively [24]. A well-characterized cellulolytic bacterium, *Serratia marcescens*, can hydrolyze lignocellulose, contributing to the breakdown of plant biomass [25]. *Brevundimonas vesicularis* exhibits cellulase production and plays a role in lignocellulose decomposition [26]. These enzymatic activities accentuate the potential of these bacteria to enhance lignocellulose degradation in AD systems, thereby improving process efficiency and biogas yields.

The intrusive growth and potent fiber-degrading enzymes of fungi including anaerobic fungi (AF) are crucial in the degradation of complex organic compounds. Anaerobic fungi play a critical role in fiber degradation in the gut of herbivores. The incorporation of animal-based substrates has given rise to AF in anaerobic digesters as AF are an essential part of herbivores’ manure [18]. However, the survival of AF in such anaerobic environments relies on the operational conditions of the digesters, including but not limited to temperature and retention time. Some studies have established the implications of elevated temperature and extended retention time on the proliferation and survival of cultivable and non-cultivable AF in anaerobic digesters [27,28]. While elevated temperatures can improve the metabolic activities of some anaerobic fungi, extended retention times promote the adaptation and thriving of anaerobic fungi in anaerobic digesters. Combinations of these parameters contribute to the metabolism of organic materials, thus improving digester performance [29]. Since microbial communities, including fungi, play a key role in these metabolic processes, and fungal community shifts have been implicated in the kinetics of AD as well methane yield, understanding their dynamics is essential for improving the efficiency of biogas production [30]. This would pave the way towards possibly optimizing the AD process and enhancing the synergistic interactions between inoculated bacteria and the indigenous fungal community of anaerobic digesters. To understand the dynamics of environmental fungal communities, which include AF, employing a metagenomic approach provides insights into the taxonomic diversity and genetic potential of fungal communities of complex samples like anaerobic digestate [30,31,32]. Determining fungal community shifts in anaerobic digesters bioaugmented with bacteria is important for understanding the resilience and adaptation of fungi to environmental changes under certain conditions such as bioaugmentation. This study deals with an in-depth evaluation of fungi as a distinct ecological guild within anaerobic digesters bioaugmented with distinct bacterial species. It also evaluates the dynamics of the dominant fungal community structure of bioaugmented anaerobic digesters through DNA metabarcoding, to provide insight into the predicted functional abilities of the fungal microbiome of anaerobic digesters. Anaerobic digestion provides a model for studying these shifts, and insights into fungal dynamics during biogas production could help in the development of strategies for more stable and sustainable biogas systems.

## 2. Materials and Methods

### 2.1. Sampling

Water hyacinth was harvested from Hartbeespoort Dam in the North West province (25°44′51″ S 27°52′1″ E), South Africa, and fresh cow dung was randomly collected from the dairy parlor of the Agricultural Research Council—Animal Production in Gauteng province (25°53′59.6” S 28°12′51.6” E), South Africa, using a clean trowel and transferred to anaerobic bags. The sample characteristics were, for water hyacinth, dry matter: 5.97%, volatile solids: 4.46%, pH: 8.11, carbon-to-nitrogen ratio: 14.5; for cow dung, dry matter: 16.8%, volatile solids: 14.16%, pH: 8.34, carbon-to-nitrogen ratio: 23.7. Samples were transported (cow dung in anaerobic bags) to the biogas laboratory of the Agricultural Research Council—Soil Climate and Water for storage. Water hyacinth was stored at −20 °C and cow dung was stored in an air-tight container at 4 °C until usage.

Ten sets of treatments were assembled in 500 mL Schott batch culture bottles fitted with screw caps, each with a working volume of 250 mL. The solid biomass consisted of freshly chopped water hyacinth cut into 2 cm × 2 cm pieces and cow dung at 2% total solids mixed in a 2:1 ratio. The sets differed for the inoculum (OD_600_ 1.5 about 10^9^ CFU/mL) consisting of 5% (*v*/*v*) of a previously identified and tested single pure bacterial culture [20]. In the first eight sets, a single pure culture (OD_600_ 1.5 about 10^9^ CFU/mL) of the mentioned strains (Table 1) was added, and another set was inoculated with a mix of the previous strains (each of the strains had OD_600_ 1.5 about 10^9^ CFU/mL), i.e., the ‘CONS’ treatment. To check the influence of living bacterial inocula on the biogas production, the negative control ‘CONT’ (tenth set), without inoculated bacteria (CONT), was set up (Table 1). The volume of each treatment was bulked to 250 mL with tap water. The treatments were not purged with nitrogen gas to create a conducive environment for the methanogens; however, anaerobiosis was reached by allowing treatments to incubate in sealed screw-capped 500 mL Schott batch culture bottles which prevented oxygen infiltration and promoted consumption of residual oxygen by microbial activity. The experiment was conducted in triplicates. The treatments were subjected to AD at a mesophilic temperature of 30 °C and 120 rpm for a period of 35 days, during which biomethane production was monitored using a Gas Chromatograph (SRI 8610C, CHROMSPEC Canada). The results of the methane/biogas production as well as the dynamics of the bacterial and archaeal (*16S rRNA* gene) communities are outlined by Obi et al. [20].

### 2.2. Genomic DNA Extraction from Different Bioaugmented Treatments

Samples were collected from each treatment, including the control, before and after AD to explore the fungal community structure of the anaerobic digesters. Following the method outlined by Mukhuba et al. [33] with modifications, a 2 mL volume of pooled digester samples (composite samples) was centrifuged at 10,000× *g* for 3 min to collect the cells, and the pellets that settled at the bottom of the tube were used for genomic DNA extraction. Genomic DNA was extracted from the collected samples using the DNeasy PowerSoil kit (Qiagen, Carlsbad, CA, USA) as per the manufacturer’s instructions and the isolated DNA was standardized to a concentration of 5 ng/µL using a Qubit 2.0 fluorometer (Invitrogen, Carlsbad, CA, USA).

#### 2.2.1. Preparation of Internal Transcribed Spacer (*ITS*) Gene Library and Analysis

To perform taxonomic profiling of the fungal *ITS2* region, a set of primers, *ITS3* (5′-CAHCGATGAAGAACGYRG-3′) and *ITS4* (5′-TTCCTSCGCTTATTGATATGC-3′), was used [34,35,36]. The 5′-end of each primer was attached with Illumina overhang adapters (TCGTCGGCAGCGTCAGATGTGTATAAGAGACAG and GTCTCGTGGGCTCGGAGATGTGTATAAGAGACAG) (Illumina, Inc., San Diego, CA, USA). Amplification via PCR was performed using 12.5 ng of DNA template, 12.5 µL of One Taq 2X Master Mix with Standard Buffer (MO482S, New England Biolabs. Inc. Ipswich, MA, USA), 0.2 µM (1 µL) of each of the primers, and PCR-grade water in a 25 µL reaction. The PCR was conducted in a Bio Rad thermal cycler (Bio-Rad Laboratories, Hercules, CA, USA) with the following conditions: initial denaturation at 94 °C for 30 s followed by 35 cycles of 94 °C for 30 s, 65 °C for 30 s, and 68 °C for 1 min, with a final extension at 68 °C for 5 min. The amplicons were confirmed on 1% agarose gel, and preparation of amplicon libraries was carried out according to Illumina’s protocol (Illumina, Inc., San Diego, CA, USA) for 2 × 300 bp paired-end reads. Sequencing was performed using the Illumina MiSeq sequencer (Illumina, Inc., San Diego, CA, USA) at the Agricultural Research Council—Biotechnology Platform (ARC—BTP), Pretoria, South Africa. Subsequent to adapter and primer sequence trimmings, raw fastq ITS sequences were obtained and subjected to bioinformatics analyses.

#### 2.2.2. Bioinformatics Analyses for Fungal Community in Anaerobic Digesters

In analyzing the fungal *ITS* sequences, an automated PIPITS pipeline was utilized [37]. A tab-delimited text file was generated for all paired-end reads from the raw sequence directory using PIPITS_GETREADPAIRSLIST. PIPITS-PREP activated its action by joining the paired-end sequences on the overlapping regions with PEAR [38], and the FAST QUALITY FILTER FASTX-Toolkit was employed to quality-filter the sequences [39]. A total of 1,667,405 reads were produced from all the treatments (20 samples) for the PIPITS_prep study. Of these, 1,320,289 reads were joined, and 1,305,701 reads passed the quality filter. To eliminate redundancy, representative sequences were obtained and the *ITS2* sub-region was extracted from 1,198,541 sequences with the aid of ITSx [40]. Reads that did not contain an *ITS* region were discarded. Short sequences (<100 bp) were also removed during processing, and the resulting sequences were clustered into 1654 operational taxonomic units (OTUs) and 454 phylotypes at a 97% sequence similarity threshold. De novo VSEARCH was used to detect and remove chimeras from the representative sequences [41]. Taxonomic assignment to representative sequences was performed using the Ribosomal Database Project (RDP) Classifier (version 2.1) [42] against the UNITE UCHIME fungal ITS reference dataset (version 7.1) [43,44,45]. Prior to analysis, the generated fungal taxonomic data were normalized to 63056 sequences per sample using the median sequencing depth. The fungal ecological guild at the genus taxonomic rank was envisaged using FUNGuild, an open annotation tool (http://www.stbates.org/guilds/app.php [46] (accessed on 1 March 2020)). Correlograms were generated using the Hmisc (v. 4.4-2), Corr (v.0.4.3), and Corrplot (v. 0.84) packages of R software. Correlation analysis was based on the Spearman rank correlation coefficient. The correlation matrix was reordered according to the correlation coefficient using the “hclust” method. Correlation network analysis was generated using the phyloseq (v.1.38.0), dyplr (v.1.1.2), and ggplot2 (v.3.4.4) packages of R software. The threshold was set at 0.3 to include only significant correlations in the network.

#### 2.2.3. Data Availability

The raw sequences generated from this study have been submitted to the Sequence Read Archive (SRA) at NCBI as part of the BioProject PRJNA704473 (https://www.ncbi.nlm.nih.gov/sra/PRJNA704473, accessed on 1 March 2020), as an experiment entitled “Dynamics of fungal community structure during biogas production”. These sequences can be accessed through the biosample accession numbers from SAMN18042783 to SAMN18042802, as well as the SRA accession numbers from SRR13779324 to SRR13779343.

### 2.3. Statistical Analysis

The fungal community structure was examined using non-metric dimensional scaling (NMDS) in multivariate spaces. Differences among treatment groups in these spaces were evaluated based on the Bray–Curtis dissimilarity. All statistical analyses were performed using R software (v.4.03) and Excel 2013 unless stated otherwise. Construction of NMDS was executed with the vegan (v. 2.5.7), ggplot2 (v. 3.4.4), and dyplr (v. 1.1.2) packages of R software (https://cran.r-project.org/, accessed on 1 March 2020).

## 3. Results

### 3.1. Batch Culture Analysis for Methane Production

**Figure 1:**
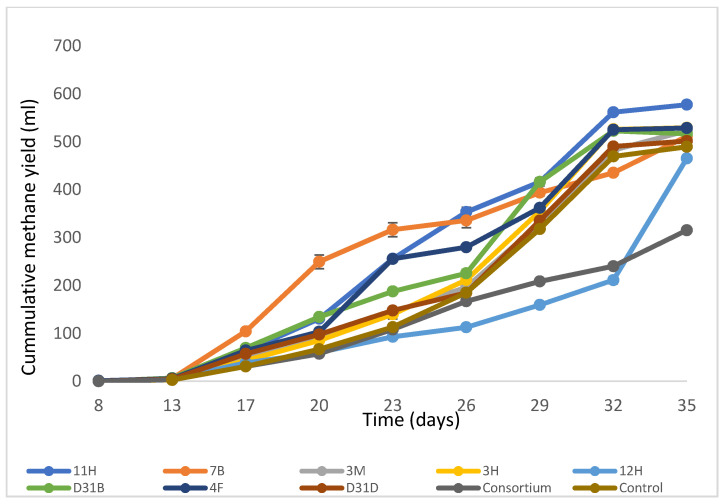
Cumulative methane yield of AD of water hyacinth and cow dung inoculated with pure cultures (adapted from [20].

The results of the methane/biogas production as well as the dynamics of the prokaryotic (bacteria and archaea) communities are detailed in Figure 1 [20]. The study investigated the effects of bioaugmentation on the diversity of the prokaryotic community structure in anaerobic digesters. It was found that the treatment inoculated with 11H (*Serratia marcescens*) produced the highest cumulative methane yield of 0.68 L, representing a 45.6% increase compared to the consortium treatment, which produced the lowest methane yield (Figure 1).

However, bioaugmentation had a negligible effect on the overall prokaryotic community structure. The bacterial community, initially dominated by the genus *Pseudomonas*, shifted to *Bacteroides* after AD. No archaeal community was detected before AD, but after AD, *Methanosarcina* dominated the archaeal communities across all treatments.

### 3.2. High-Throughput Metabarcoding of ITS Genes from Different Bioaugmented Treatments

**Figure 2:**
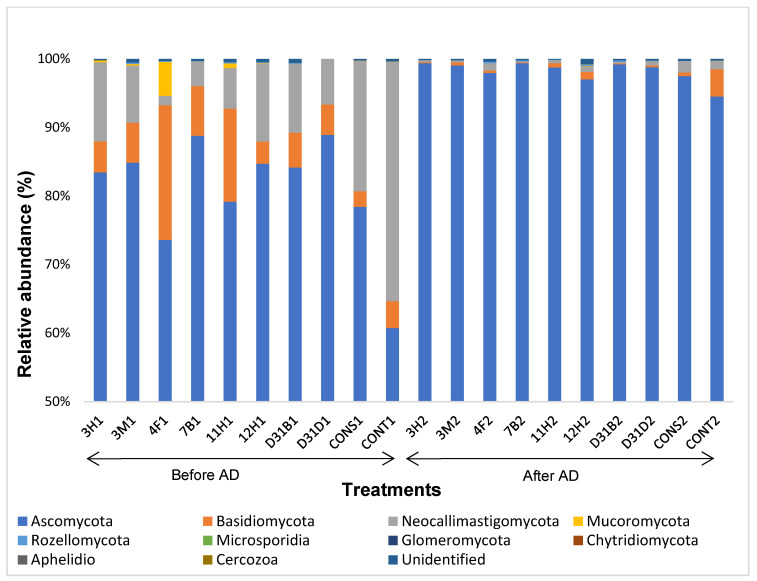
Relative abundance (≥1%) of fungal phyla in treatments before AD and after AD. Phylotypes with an average relative abundance of less than 1% and those unclassified at the phylum level were excluded from the plot.

Variations in taxonomic classification at the phylum, class, and genus levels were observed within the fungal community of anaerobic digesters subjected to bacterial bioaugmentation. The relatively abundant fungal phyla, including Ascomycota, Basidiomycota, and Neocallimastigomycota, were identified across all treatments. Notably, Ascomycota accounted for 60–99% of the relatively abundant sequences within the fungal phyla, with increased dominance observed in most treatments after AD (Figure 2). Ascomycota exhibited a remarkable dominance, constituting approximately 99% of the fungal composition in about 60% of the treatments after AD. This prevalence was particularly evident in treatments bioaugmented with specific bacterial strains such as *Pseudomonas stutzeri* (3H2), *Exiguobacterium mexicanum* (3M2), *Lysinibacillus fusiformis* (7B2), *Serratia marcescens* (11H2), *Acinetobacter iwoffii* (D31B2), and *Planococcus maritimus* (D31D1). In these treatments, the relative abundance of Ascomycota ranged from 11% (before AD) to 25% (after AD).

Contrastingly, the control treatment (CONT2), which was not bioaugmented, recorded a 54% difference (increase) in the relative abundance of Ascomycota after AD. This underscores the impact of bacterial bioaugmentation on shaping the taxonomic dynamics of fungal communities in anaerobic digesters. This observation highlights the importance of bacterial bioaugmentation as a tool for influencing the microbial community structure of anaerobic digesters. The shift suggests that bacterial bioaugmentation probably suppressed some fungal populations by reducing their reliance on fungal-associated hydrolysis, thus leading to bacterial-dominated hydrolysis. In addition to the control treatment CONT2, the remarkable presence of Basidiomycota was noted only in treatment 12H2 (post-AD). The presence of Neocallimastigomycota and Basidiomycota markedly decreased after AD, except in the case of the control treatment (CONT2). In CONT2, the abundance of Basidiomycota remained remarkably consistent after AD, whereas Neocallimastigomycota decreased by 97%. The substantial reduction in the presence of Neocallimastigomycota, a notable anaerobic fungus, in the same treatment after AD might be attributed to the absence of bioaugmented bacteria.

**Figure A1:**
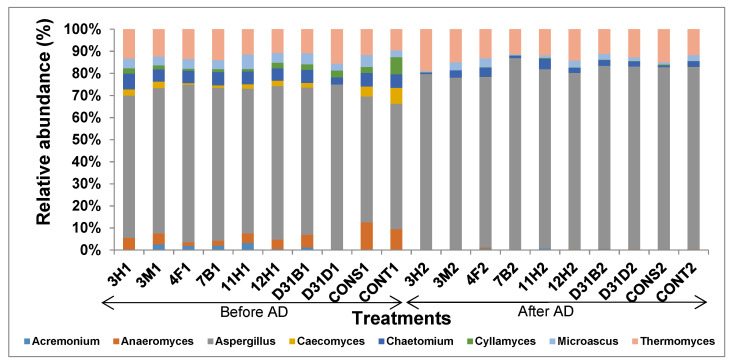
Relative abundance of dominant fungal class in treatments before and after batch anaerobic digestion. Unclassified phylotypes at the class level were omitted.

Examining the class level, the prevalent fungal classes across all treatments exhibit a similarity in composition to the taxonomic ranking at the phylum level. The dominant classes include Eurotiomycetes, Sordariomycetes, and Dothideomycetes, along with Tremellomycetes, with Eurotiomycetes (Appendix A, Figure A1) standing out as the most abundant class in the bioaugmentation process of anaerobic digesters across all treatments, both before and after AD. Neocallimastigomycetes, abundant in treatments before AD, showed a decrease in abundance after AD, a trend also observed in Tremellomycetes. Conversely, the appearance of Leotiomycetes after AD was observed in all treatments except 3M2 and 12H. Saccharomycetes were exclusively observed in treatment 3H2 after AD, while Agaricomycetes were present only in the control treatment CONT1 (before AD), and exhibited an increase in CONT2 (after AD).

**Figure 3:**
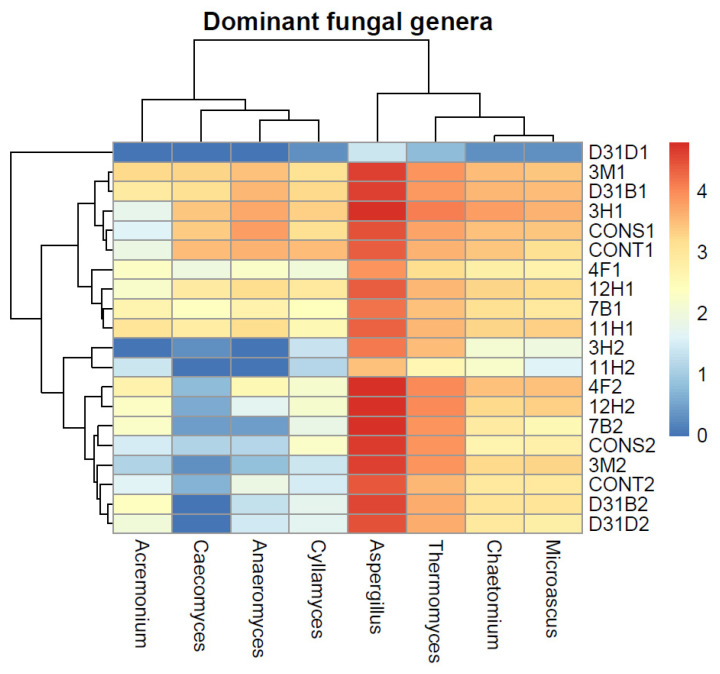
Relative abundance of dominant (≥1%) fungal phylotypes at the genus taxonomic rank before AD and after AD. Phylotypes with an average relative abundance of less than 1% and those unclassified at the genus level were excluded from the plot. The plot was generated using the average relative abundance for each group.

Further examination of fungal genera suggests that AD did not impact the abundance of obligate aerobic fungi such as *Thermomyces, Acremonium, Aspergillus, Chaetomium*, and *Microascus*. However, there was a decrease in the prevalence of anaerobic fungal genera such as *Anaeromyces, Cyllamyces*, and *Caecomyces* following AD, except in treatment D31D1. This is evident from the dominance of *Aspergillus* observed in all treatments throughout the AD period, as depicted in Figure 3 and Appendix A. The presence and absence of the aforementioned fungal genera align with the observed patterns at the phylum and class taxonomic levels, particularly concerning Necallimastigomycota and Neocallimastigomyces. Sordariomycetes and Dothideomycetes were relatively dominant across the treatments, further highlighting the importance of these fungal classes in AD (Appendix B).

**Figure 4:**
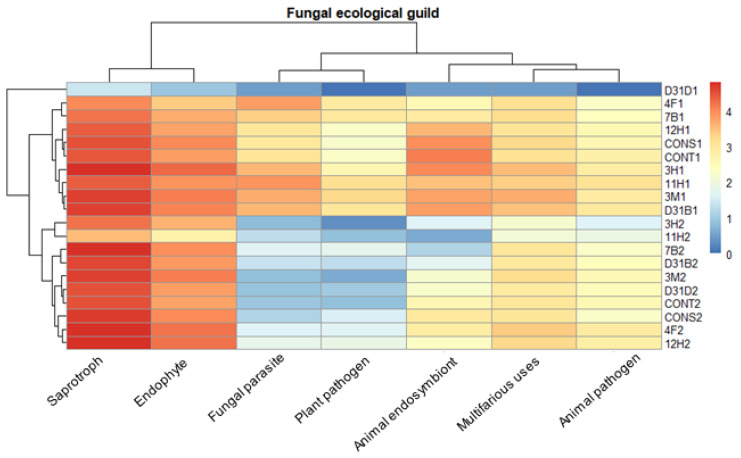
Ecological guild of fungi. Ecological guild was generated with FUNGuild database. Guild allotted as ‘multifarious uses’ comprises fungal sequences that were allotted as animal pathogens, plant pathogens, endophytes, and saprotrophs.

The majority of fungi that belong to the ecological guild are mainly saprotrophs and endophytes, as depicted in Figure 4, and were abundant before and after AD. Fungal parasites and plant pathogens, which were abundant before AD, decreased after AD in all treatments except for treatment D31D1. Treatment D31D1 had the lowest abundance of fungi, and the guild of fungi classified as animal pathogens showed similar levels of abundance across all treatments as no major change was observed before and after AD.

**Figure 5:**
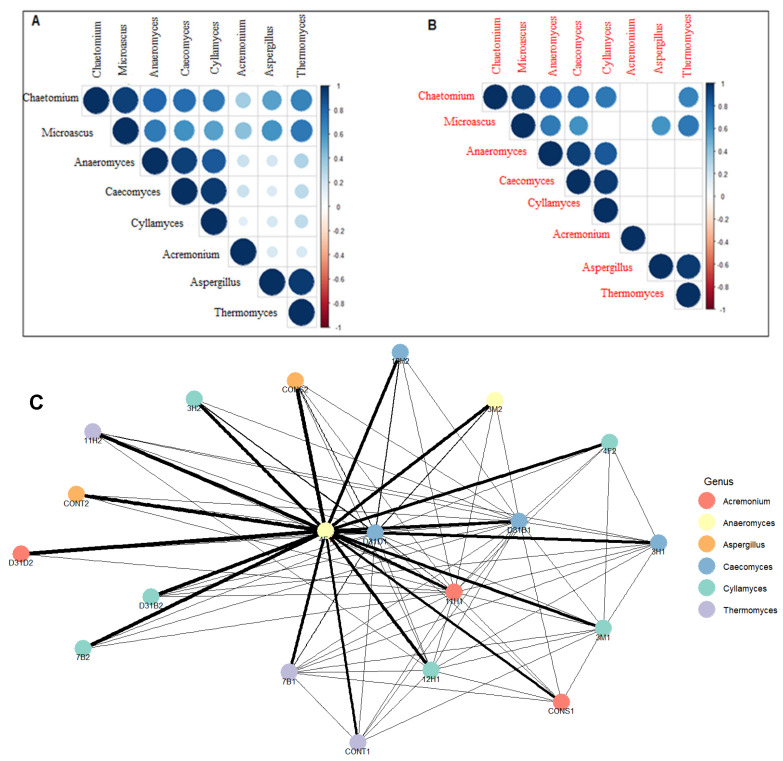
Correlation: (**A**) Correlogram showing the correlations among the dominant fungal genera. (**B**) Correlogram showing significance test for correlated fungi. (**C**) Correlation network analysis of fungal communities visualized at genus level.

The color intensity and the size of the circles (Figure 5A) are proportional to the correlation coefficients. A positive correlation is denoted by +1 or values closer to +1, while −1 shows strong negative correlations. *Chaetomium* displayed strong positive correlations with *Microascus* and positive correlations with *Anaeromyces, Caecomyces, Cyllamyces*, and *Thermomyces*. In the same vein, *Thermomyces* showed strong positive correlations with *Aspergillus* and correlated positively with a few dominant fungi (*Chaetomium* and *Microascus*) (Figure 5A). *Anaeromyces* correlated strongly with *Caecomyces* and *Cyllamyces*. Positive correlations indicate relative abundance. Correlations with a *p*-value < 0.01 were considered statistically significant and are presented as blue circles, while correlation coefficient values that are insignificant are left blank (Figure 5B). In the correlation network (Figure 5C), only correlations with a correlation coefficient of 0.3 or higher are considered significant and are included in the network. The yellow-colored *Anaeromyces* in treatment 4F1 is more central in the network with thick edges (Figure 5C), indicating multiple stronger connections with other fungal genera, especially *Acremonium* in 11H. *Acremonium*, on the other hand, showed fewer connections, which indicates a limited association with other genera. The thick edges (lines) show short ecological distances, while longer distances (thin lines) reflect greater dissimilarity, i.e., weaker correlations.

**Figure 6:**
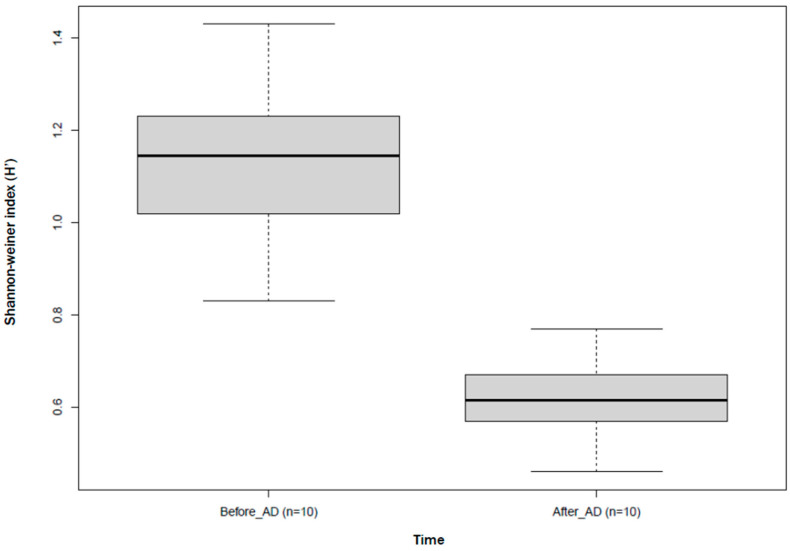
Description of fungal community diversity with Shannon–Weiner index.

The description of fungal richness and diversity showed that the richness of the fungi decreased after AD. However, some dominant genera such as *Cyllamyces*, *Caecomyces*, *Anaeromyces*, and *Acremonium* decreased after AD, corresponding to the information in Figure 2 and Appendix A. A decrease in the fungal community diversity was observed after AD (Figure 6) and the Shannon–Weiner index (H′) was used to measure the diversity within the fungal community, incorporating both species richness and evenness. It indicated moderate diversity, suggesting a more even distribution of fungi before AD (Figure 6). The after-AD treatments showed low diversity in composition as just a few fungi dominated the fungal community after AD, with the majority of fungi belonging to particular phyla, classes, or genera.

**Figure 7:**
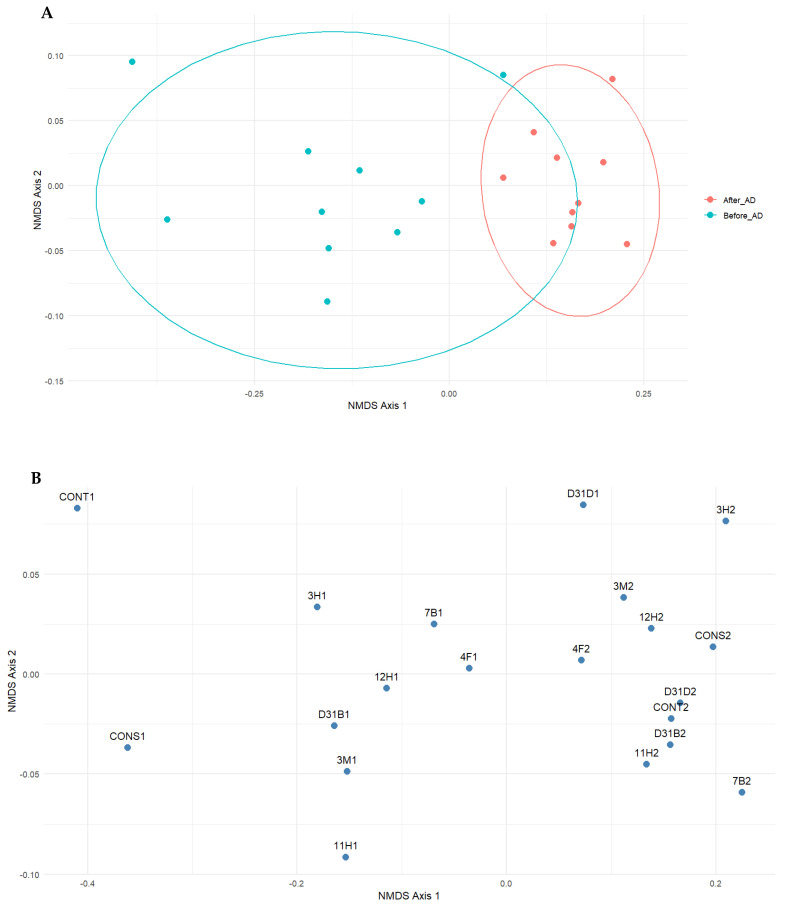
Bray–Curtis dissimilarity between fungal communities at the genus taxonomic rank. (**A**) NMDS to envisage the multivariate structure of the fungal communities at the genus taxonomic rank before and after AD. (**B**) NMDS showing the treatments the fungi belong to. The stress value of the NMDS plot is 0.03 (Appendix C).

Communities with similar fungi were clustered together. Multivariate ordination methods explored the fungal community structure. Treatments that are closer together have comparable fungal structures (Figure 7), indicating the ecological distances between them. A separation of clusters between the treatments before AD and after AD was observed. However, treatment D31D1 clustered with the after-AD treatments, indicating similarity in their fungal diversity (Figure 7). The stress value of the non-metric multidimensional scaling (NMDS) plot, depicted in Figure 7, is 0.03. The stress plot, which provides additional information, can be referred to in Appendix C.

**Figure 8:**
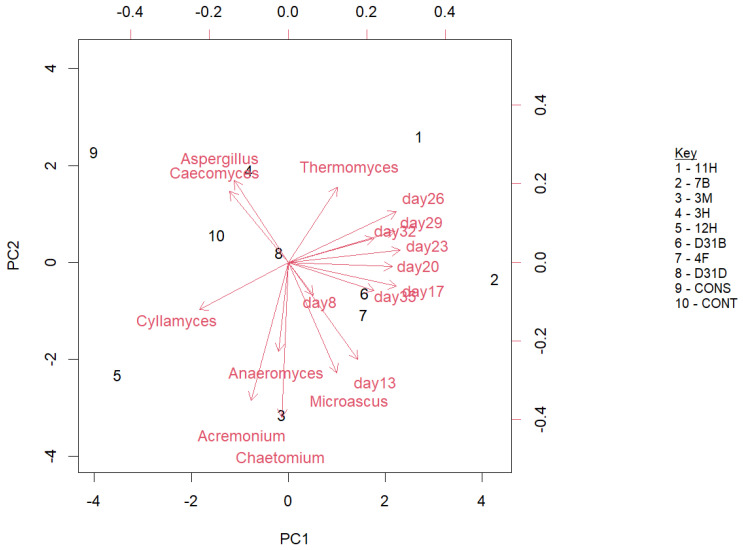
Principal component analysis (PCA) to envisage relationship between anaerobic digestion dynamics and fungi at genus taxonomic rank over time.

Insights into the fungal genera and AD dynamics over time suggest a change in the composition of the fungal community over time (days). In Figure 8, Aspergillus and Caecomyces are positive associated with PC2 (*y*-axis), while Cyllamyces is negatively linked to PC2 (negative scores). Chaetomium and Acremonium are located closer to the origin, indicating weaker contributions compared to other genera. The top contributors to PC1 include day 23, day 26, and day 29, while the top contributors to PC2 are day 9 and day 32.

## 4. Discussion

There are limited studies on the core fungal community structures in bacterial-bioaugmented anaerobic digesters, despite fungi being an essential part of the microbiota involved in the anaerobic digestion of lignocellulosic substrates. In this study, the structure and composition of the basic fungal community of different bacterial-bioaugmented anaerobic digesters were investigated by sequencing of the *ITS* region. Presently, the *ITS* region has the greatest number of reference sequences in the GenBank and the use of *ITS2* as a molecular marker stems from its conservation within fungi. However, the existing reference sequences in the public database have a low rate of sequence identification for basal fungal lineages, making some taxa unrepresentative in the reference database [12,47].

Findings from the present study highlight the specific fungal taxa that thrive in the anaerobic digestion environment and suggest their potential significance in this process. Ascomycota was the dominant fungal phylum across all treatments. This observation aligns with the findings of Sun et al. [48], who also reported the predominance of Ascomycota-associated fungi in anaerobic digesters. Neocallimastigomycota was relatively abundant before AD but decreased after AD, and a similar trend was also observed for Basidiomycota. The decrease in the abundance of Neocallimastigomycota, a key anaerobic fungus, in the same treatment after AD could be attributed to a shift in the native anaerobic fungal community, which could be a response to changes in environmental or operational conditions and metabolic by-products as well as substrate availability [27,28]. Several studies have shown Neocallimastigomycota to be the predominant anaerobic fungal phylum [12,49,50]. The fungi in this group are known as obligate anaerobes, and their high abundance before AD is likely because their natural habitat is in the digestive system of herbivores such as cows [12,51]. This corresponds with the findings of Zhang et al. [52], who reported a decreased abundance of Neocallimastigomycota in the ruminal microbiota of ruminants upon the inclusion of lignocellulosic materials in their diet. However, it contradicts the findings of Langer et al. [2], as the phylum Neocallimastigomycota was absent in the analyzed anaerobic digesters with cow dung as part of the substrates. Neocallimastigomycota has been reported to degrade lignocellulose while co-existing with bacteria and methanogens during anaerobic digestion for the production of biogas [53]. The fungal phylum Basidiomycota was dominant before AD but reduced after AD in most treatments; however, the prevalence of Basidiomycota in the control treatments, both before and after AD, might be due to the lack of inoculated bacteria, thus affecting the fungal community dynamics in bioaugmented anaerobic digesters. Eurotiomycetes emerged as the dominant fungal class both before and after AD, indicating their resilience and adaptability within the system. Sordariomycetes and Dothideomycetes were relatively dominant across the treatments, further highlighting the importance of these fungal classes in AD. The dominant genera (which belonged solely to Ascomycota and Neocallimastigomycota) across all treatments included *Aspergillus*, *Thermomyces*, *Chaetomium*, and *Microascus*. However, this contrasts with the findings of Dollhofer et al. [49], who reported different genera, *Neocallimastix* and *Piromyces*, to be the most abundant genera in anaerobic digesters that had substrates such as sugar beets, silage grass, etc., and the digestion temperature ranged between 38 and 53 °C. Although there exists a similarity to this study in terms of the inclusion of substrates such as cow dung, the present study showed the relative abundance of *Neocallimastix* and *Piromyces* to be less than 1%. The reduced abundance of anaerobic fungal genera (*Anaeromyces, Cyllamyces, Caeomyces*) after AD may suggest limited substrate availability or a shift in microbial interactions within the AD environment. This shift in fungal composition suggests a dynamic response to the AD process.

The majority of fungi, as indicated by the ecological guild classification (Figure 4), were identified as saprophytes (saprotrophs), and their presence in anaerobic digesters has been previously studied [54]. The abundance of saprophytic fungi in the anaerobic digesters signifies the abundance of a diverse range of enzymes and subsequently enhanced organic matter decomposition. This highlights the functional alignment of fungal communities (saprophytes) for the degradation of organic matter. Saprophytes contribute to the stability of the AD process by promoting a balanced microbial community as they work in synergistic interaction with prokaryotic communities in anaerobic digesters [55]. *Aspergillus* sp., being saprophytes, demonstrated dominance across all treatments during AD. The abundance of saprophytes in the digester may not directly correlate with their metabolic efficiency since saprophytes typically thrive in aerobic environments. This is evident in the cumulative methane yield from the anaerobic co-digestion of water hyacinth and cow dung inoculated with pure bacteria isolates, as reported by Obi et al. [20]. The abundance of *Aspergillus* across all treatments suggests their potential to utilize nitrate as an oxidant in an ATP-generating process during AD. This allows for their proliferation but not optimal growth under oxygen-limited conditions [56,57]. Liu et al. [58] outlined the ability of different species of *Aspergillus* to produce different types of hydrolases that catalyze the rate-limiting phase (hydrolysis) of the AD of the lignocellulosic substrates. This further confirms their presence and potential activities in anaerobic digesters. However, the drastic reduction in their community diversity (Figure 7) after AD could be related to the limited oxygen concentration of the anaerobic digesters. Another guild that exhibited relatively high abundance after AD was the endophytes, which are non-pathogenic microorganisms. Their presence in the potential digestate indicates its suitability for use as a soil ameliorant. The reduced abundance of fungal parasites and pathogens in the treatments following AD suggests the potential benefits of AD in effectively mitigating or suppressing the presence of these pathogenic organisms, making the resulting digestate a safer option for utilization as a possible soil ameliorant. However, the resilience of the animal pathogen guild across treatments is a concern in managing zoonotic risks relating to waste management and agriculture. Only a few recent studies have explored fungi in anaerobic reactors [3,4,27,49]. To the best of our knowledge, this is one of the first studies to characterize the ecological guilds of fungal communities in bacteria-bioaugmented anaerobic digesters focusing on their potential suitability for agricultural and environmental applications.

**Figure A5:**
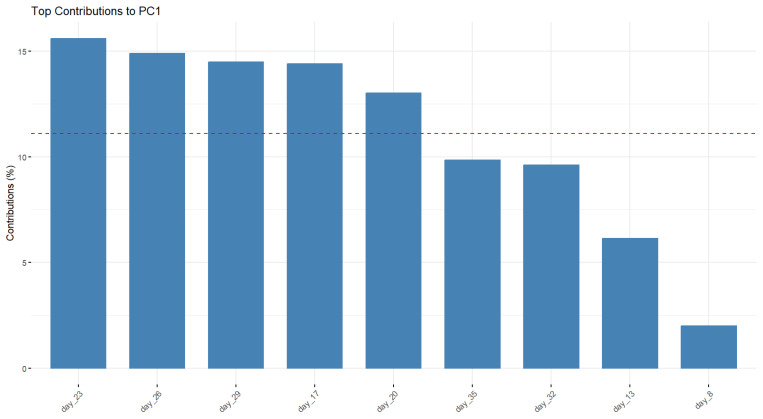
Top contributions of samples (days) to the second principal component (PC1) from the principal component analysis (PCA).

**Figure A6:**
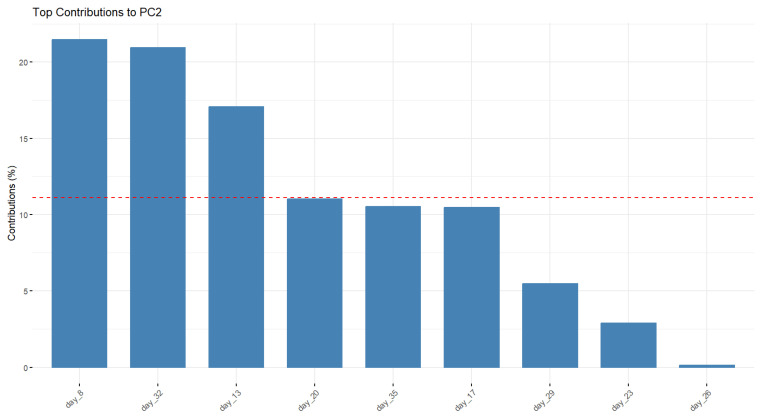
Top contributions of samples (days) to the second principal component (PC2) from the principal component analysis (PCA).

The correlograms presented in Figure 5 offer a comprehensive visualization of the correlation patterns within the dataset. The predominance of positive correlations between *Thermomyces* and other dominant fungi is a key finding that suggests potential ecological and functional relationships. This analysis contributes to our understanding of the relationship between microorganisms in this specialized ecosystem and highlights areas for further exploration and research. The strong connection (thick lines in the correlation network analysis, Figure 5C) of fungal genus *Anaeromyces* in treatment 4F1 indicates ecological distances from other genera, specifically *Acremonium* in 11H. A short ecological distance and strong positive correlations (as indicated by the thick lines) portray the ability of the organisms involved to co-occur and respond to similar environmental conditions. The genera connected by thick lines are the key players within the network, likely exerting greater influence on the community dynamics than others due to their strong interactions. The diversity indices used to assess fungal richness and diversity revealed a decrease in both measures following AD, indicating a negative impact of AD on fungal communities. This observation suggests that the AD process may favor certain fungal taxa. This could affect the overall functional potential of the fungal microbiome. The treatment bioaugmented with 4F1 (Bacillus cereus) exhibited the most connections to other treatments. Although genera such as *Caecomyces* and *Cyllamyces* are connected to multiple treatments, their thin edges indicate a weak association with other linked genera. Treatments CONT2 and D31D1 showed the presence of different fungal communities due to their multiple connections, although these connections were weak. Less connected genera like *Thermomyces* and *Acremonium* show unique fungal taxa that thrive under special ecological conditions. The multivariate ordination methods applied in this study suggest a comprehensive view of the fungal community structure and its dynamics before and after AD. The observed clustering patterns and separation of treatments before and after AD highlight the ecological shifts that occur during this process. The clustering of treatment D31D1 (a ‘before-AD’ treatment) with the ‘after-AD’ treatments shows comparable fungi and emphasizes the ecological distances, highlighting the complexity of fungal interactions and community dynamics in specialized ecosystems like anaerobic digesters. These insights contribute to our understanding of the stability and adaptability of the fungal microbiome of anaerobic digesters and how fungal communities respond to and influence the AD process, paving the way for more targeted research into optimizing and managing these systems for various applications, including biogas production and waste management. The shift in the fungal community could be due to microbial interactions, as earlier suggested, or environmental changes in the digesters. The dominance of *Thermomyces* and *Caecomyces* during the later stages (day 26 and day 29) of AD indicated their possible metabolic roles towards the later stages of AD. The location of *Chaetomium* and *Acremonium* closer to the origin (Figure 8) shows their weaker contributions to the fungal dynamics compared to other genera. The contribution of day 23, day 26, and day 29 (Figure 1) to PC1 reflects the important stages of AD where a significant shift was observed in the digester performance or fungal community. The mid-to-late stages of AD (day 23–day 29) are fundamental to the microbial community dynamics that drive the variability in PC1 (Appendix E, Figure A5). This indicates periods of optimal changes in the digester. Day 9 and day 32 contributed strongly to PC2, indicating their link to distinct fungal community shifts, while low contributors to PC2 include days 29, 23, and 26 (Appendix F, Figure A6).

## 5. Conclusions

The findings of this study describe the distribution of fungi as well as the ecological guild, thereby contributing to our understanding of the community structure of the fungal ecosystem of anaerobic digesters. They also reflect the resilience and adaptability of Ascomycota and their ability to thrive in an unfavorable environment despite their ecological nature. The relative increase in Ascomycota after AD in non-bioaugmented digesters shows the potential of bacteria bioaugmentation in modeling the fungal community dynamics of anaerobic digesters. However, further research could employ absolute quantification methods such as qPCR to track changes in fungal population sizes over time. The study also revealed additional fungal phyla, beyond the well-known anaerobic Neocallimastigomycota, within anaerobic digesters. These findings expand our knowledge on the use of high-throughput metabarcoding approaches to explore the microbial ecology in specialized ecosystems, with implications for optimizing AD processes and harnessing the full potential of these systems for biogas production and waste management. However, a limitation of this study was that fungal community analysis was performed on pooled samples from three replicated digesters rather than each replicate separately. While the *ITS* region is widely used for fungal identification and phylogenetic studies, it is a possible limitation in this study as targeting other regions such as 28S rRNA or 18S rRNA could provide more information in the fungal community analysis. Exploring synergistic cross-kingdom interactions between bacteria and fungi is a major perspective for fungal research in AD field. This includes further investigation of fungal-driven pretreatment of lignocellosic substrates to enhance its AD for optimal biogas production. Incorporation of transcriptomic or proteomics approaches will give precision to improved strategies for enhancing the efficiency and stability of AD systems while promoting sustainable bioenergy production. Further investigation could focus on the effect of a different potential bacterial/fungal inoculum on different substrates.

## Figures and Tables

**Table 1:** Bacterial isolates’ ID and bioaugmentation treatments’ composition.

Treatment ID Before AD	Treatment ID After AD	Inoculated Bacteria with GenBank Accession Numbers	Treatment Make-Up
3H1	3H2	*Pseudomonas stutzeri* (MK104459)	WH + CD + 10^9^ cfu/mL of 3H
3M1	3M2	*Exiguobacterium mexicanum* (MK104464)	WH + CD + 10^9^ cfu/mL of 3M
4F1	4F2	*Bacillus cereus* (MK104469)	WH + CD + 10^9^ cfu/mL of 4F
7B1	7B2	*Lysinibacillus fusiformis* (MK104485)	WH + CD + 10^9^ cfu/mL of 7B
11H1	11H2	*Serratia marcescens* (MK104517)	WH + CD + 10^9^ cfu/mL of 11H
12H1	12H2	*Brevundimonas vesicularis* (MK104523)	WH + CD + 10^9^ cfu/mL of 12H
D31B1	D31B2	*Acinetobacter iwoffi* (MK104525)	WH + CD + 10^9^ cfu/mL of D31B
D31D1	D31D2	*Planococcus maritimus* (MK104526)	WH + CD + 10^9^ cfu/mL of D31D
CONS1	CONS2	*Pseudomonas stutzeri* (MK104459), *Exiguobacterium mexicanum* (MK104464), *Bacillus cereus* (MK104469) *Lysinibacillus fusiformis* (MK104485), *Serratia marcescens* (MK104517), *Brevundimonas vesicularis* (MK104523), *Acinetobacter iwoffi* (MK104525), and *Planococcus maritmus* (MK104526)	WH + CD + 10^9^ cfu/mL of 3H + 10^9^ cfu/mL of 3M + 10^9^ cfu/mL of 4F + 10^9^ cfu/mL of 7B + 10^9^ cfu/mL of 11H + 10^9^ cfu/mL of 12H + 10^9^ cfu/mL of D31B + 10^8^ cfu/mL of D31D
CONT1	CONT2	No inoculated bacteria	WH + CD

WH = water hyacinth; CD = cow dung.

**Figure A2:**
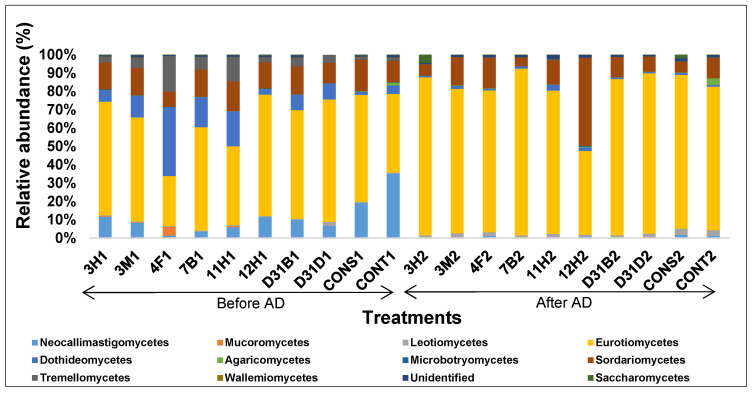
Relative abundance of dominant fungal genera in treatments before and after batch anaerobic digestion. Unclassified phylotypes at the genus level were omitted.

**Figure A3:**
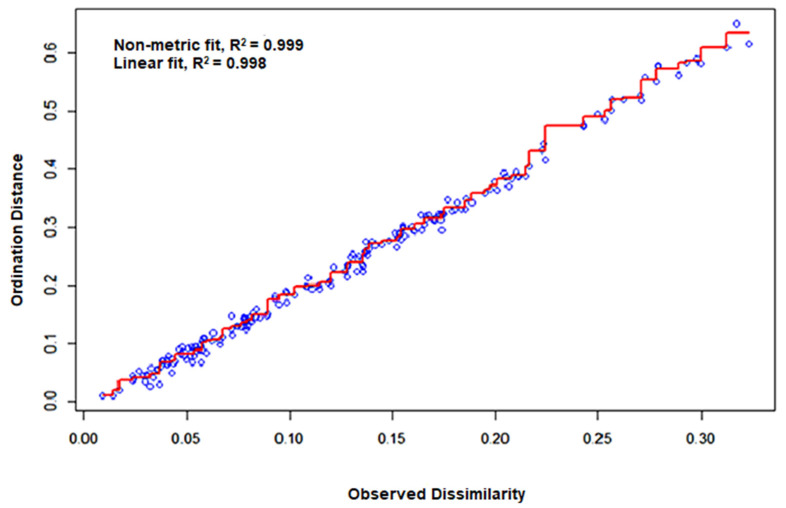
Stress plot for the NMDS plot in Figure 5. Plot was generated to ascertain the integrity of the ordination fit. The ‘stressplot’ function of the vegan package of the R software was used to create the stress plot.

**Figure A4:**
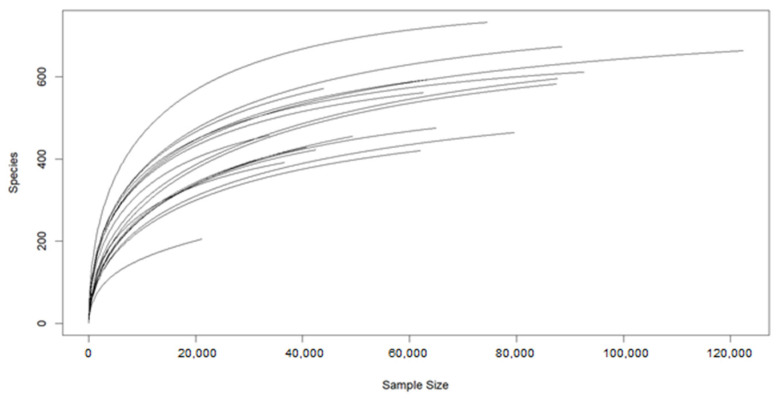
Rarefaction curve of fungi.

## Data Availability

The raw sequences are available at https://www.ncbi.nlm.nih.gov/sra/PRJNA704473 (accessed on 1 March 2020).

## References

[1] Suman A. (2021). Role of renewable energy technologies in climate change adaptation and mitigation: A brief review from Nepal. Renew. Sustain. Energy Rev..

[2] Langer S.G., Gabris C., Einfalt D., Wemheuer B., Kazda M., Bengelsdorf F.R. (2019). Different response of bacteria, archaea and fungi to process parameters in nine full-scale anaerobic digesters. Microb. Biotechnol..

[3] Young D., Dollhofer V., Callaghan T.M., Reitberger S., Lebuhn M., Benz J.P. (2018). Isolation, identification and characterization of lignocellulolytic aerobic and anaerobic fungi in one- and two-phase biogas plants. Bioresour. Technol..

[4] Yang X., Zhang Z., Li S., He Q., Peng X., Du X., Feng K., Wang S., Deng Y. (2022). Fungal dynamics and potential functions during anaerobic digestion of food waste. Environ. Res..

[5] Seppälä S., Wilken S.E., Knop D., Solomon K.V., O’malley M.A. (2017). The importance of sourcing enzymes from non-conventional fungi for metabolic engineering and biomass breakdown. Metab. Eng..

[6] Caruso M.C., Braghieri A., Capece A., Napolitano F., Romano P., Galgano F., Altieri G., Genovese F. (2019). Recent updates on the use of agro-food waste for biogas production. Appl. Sci..

[7] Ren Y., Yu M., Wu C., Wang Q., Gao M., Huang Q., Liu Y. (2018). A comprehensive review on food waste anaerobic digestion: Research updates and tendencies. Bioresour. Technol..

[8] Tian H., Yan M., Treu L., Angelidaki I., Fotidis I.A. (2019). Hydrogenotrophic methanogens are the key for a successful bioaugmentation to alleviate ammonia inhibition in thermophilic anaerobic digesters. Bioresour. Technol..

[9] Gállego-Bravo A.K., García-Mena J., Piña-Escobedo A., López-Jiménez G., Gutiérrez-Castillo M.E., Tovar-Gálvez L.R. (2023). Monitoring of a microbial community during bioaugmentation with hydrogenotrophic methanogens to improve methane yield of an anaerobic digestion process. Biotechnol. Lett..

[10] Bernardi A.V., de Gouvêa P.F., Gerolamo L.E., Yonamine D.K., Balico L.d.L.d.L., Uyemura S.A., Dinamarco T.M. (2018). Functional characterization of GH7 endo-1,4-β-glucanase from Aspergillus fumigatus and its potential industrial application. Protein Expr. Purif..

[11] Greening C., Geier R., Wang C., Woods L.C., E Morales S., McDonald M.J., Rushton-Green R., Morgan X.C., Koike S., Leahy S.C. (2019). Diverse hydrogen production and consumption pathways influence methane production in ruminants. ISME J..

[12] Vinzelj J., Joshi A., Insam H., Podmirseg S.M. (2020). Employing anaerobic fungi in biogas production: Challenges & opportunities. Bioresour. Technol..

[13] Sundberg C., Al-Soud W.A., Larsson M., Alm E., Yekta S.S., Svensson B.H., Sørensen S.J., Karlsson A. (2013). 454 pyrosequencing analyses of bacterial and archaeal richness in 21 full-scale biogas digesters. FEMS Microbiol. Ecol..

[14] Abendroth C., Vilanova C., Günther T., Luschnig O., Porcar M. (2015). Eubacteria and archaea communities in seven mesophile anaerobic digester plants in Germany. Biotechnol. Biofuels.

[15] Langer S.G., Ahmed S., Einfalt D., Bengelsdorf F.R., Kazda M. (2015). Functionally redundant but dissimilar microbial com-munities within biogas reactors treating maize silage in co-fermentation with sugar beet silage. Microb. Biotechnol..

[16] Westerholm M., Isaksson S., Karlsson Lindsjö O.K., Schnürer A. (2018). Microbial community adaptability to altered temperature conditions determines the potential for process optimisation in biogas production. Appl. Energy.

[17] Yang S., Li L., Peng X., Zhang R., Song L. (2022). Eukaryotic community composition and dynamics during solid waste decomposition. Appl. Microbiol. Biotechnol..

[18] Tsapekos P., Kougias P., Vasileiou S., Treu L., Campanaro S., Lyberatos G., Angelidaki I. (2017). Bioaugmentation with hydrolytic microbes to improve the anaerobic biodegradability of lignocellulosic agricultural residues. Bioresour. Technol..

[19] Obi L.U., Tekere M., Roopnarain A., Sanko T., Maguvu T.E., Bezuidenhout C.C., Adeleke R.A. (2020). Whole genome sequence of Serratia marcescens 39_H1, a potential hydrolytic and acidogenic strain. Biotechnol. Rep..

[20] Obi L.U., Tekere M., Roopnarain A., Adeleke R.A. Bioaugmentation Strategies to Enhance Methane Production From Lignocellulosic Substrates: Dynamics Of The Prokaryotic Community Structure. Proceedings of the 30th European Biomass Conference and Exhibition.

[21] Al Makishah N.H., Elfarash A.E. (2022). Molecular characterization of cellulase genes in *Pseudomonas stutzeri*. Electron. J. Biotechnol..

[22] Baltaci M.O., Omeroglu M.A., Albayrak S., Adiguzel G., Adiguzel A. (2022). Production of Endoglucanase by Exiguobacterium mexicanum OB24 Using Waste Melon Peels as Substrate. An. Acad. Bras. Cienc..

[23] Liao Y., Wu S., Zhou G., Mei S., Yang Z., Li S., Jin Z., Deng Y., Wen M., Yang Y. (2024). Cellulolytic *Bacillus cereus* produces a variety of short-chain fatty acids and has potential as a probiotic. Microbiol. Spectr..

[24] Biswas S., Paul D., Bhattacharjee A. (2024). Cellulolytic Potential of Lysinibacillus fusiformis Strain WGI4 Isolated From White Grub Beetle Phyllophaga sp. (Coleoptera: Scarabaeidae) Larvae Gut. Proc. Zool. Soc..

[25] Kumar H.N., Mohana N.C., Rakshith D., Abhilash M., Satish S. (2023). Multicomponent assessment and optimization of the cellulase activity by Serratia marcescens inhabiting decomposed leaf litter soil. Sustain. Chem. Pharm..

[26] Hu X., Yu J., Wang C., Chen H. (2014). Cellulolytic bacteria associated with the gut of *Dendroctonus armandi* larvae (Coleoptera: Curculionidae: Scolytinae). Forests.

[27] Dollhofer V., Dandikas V., Dorn-In S., Bauer C., Lebuhn M., Bauer J. (2018). Accelerated biogas production from lignocellulosic biomass after pre-treatment with *Neocallimastix frontalis*. Bioresour. Technol..

[28] Stoyancheva G., Kabaivanova L., Hubenov V., Chorukova E. (2023). Metagenomic Analysis of Bacterial, Archaeal and Fungal Diversity in Two-Stage Anaerobic Biodegradation for Production of Hydrogen and Methane from Corn Steep Liquor. Microorganisms.

[29] Mutungwazi A., Ijoma G.N., Matambo T.S. (2021). The significance of microbial community functions and symbiosis in enhancing methane production during anaerobic digestion: A review. Symbiosis.

[30] Miranda F.M., Azevedo V.C., Ramos R.J., Renard B.Y., Piro V.C. (2024). Hitac: A hierarchical taxonomic classifier for fungal ITS sequences compatible with QIIME2. BMC Bioinform..

[31] Sneha M.J.X., Thangavel M., Mani I., Rajapriya P., Ponnuraj N., Pandi M. (2024). Endophytic Fungal Diversity in *Hardwickia binata*: Bridging the Gap between Traditional and Modern Techniques. Microbiol. Res..

[32] Viotti C., Chalot M., Kennedy P.G., Maillard F., Santoni S., Blaudez D., Bertheau C. (2024). Primer pairs, PCR conditions, and peptide nucleic acid clamps affect fungal diversity assessment from plant root tissues. Mycology.

[33] Mukhuba M., Roopnarain A., Adeleke R., Moeletsi M., Makofane R. (2018). Comparative assessment of bio-fertiliser quality of cow dung and anaerobic digestion effluent. Cogent Food Agric..

[34] White T.J., Bruns T., Lee S., Taylor J., Innis M.A., Gelfand D.H., Sninsky J.J., White T.J. (1990). Amplification and direct sequencing of fungal ribosomal RNA genes for phylogenetics. PCR Protocols: A Guide to Methods and Applications.

[35] Tedersoo L., Bahram M., Põlme S., Kõljalg U., Yorou N.S., Wijesundera R., Ruiz L.V., Vasco-Palacios A.M., Thu P.Q., Suija A. (2014). Global diversity and geography of soil fungi. Science.

[36] Ezeokoli O.T., Mashigo S.K., Paterson D.G., Bezuidenhout C.C., Adeleke R.A. (2019). Microbial community structure and relationship with physicochemical properties of soil stockpiles in selected South African opencast coal mines. Soil Sci. Plant Nutr..

[37] Gweon H.S., Oliver A., Taylor J., Booth T., Gibbs M., Read D.S., Griffiths R.I., Schonrogge K. (2015). PIPITS: An automated pipeline for analyses of fungal internal transcribed spacer sequences from the Illumina sequencing platform. Methods Ecol. Evol..

[38] Zhang M., Sun H., Fei Z., Zhan F., Gong X., Gao S. Fastq_clean: An optimized pipeline to clean the Illumina sequencing data with quality control. Proceedings of the 2014 IEEE International Conference on Bioinformatics and Biomedicine (BIBM).

[39] Gordon A., Hannon G. Fastx-Toolkit, FASTQ/A Short-Reads Preprocessing Tools. FASTX-Toolkit. [Computer software]. https://github.com/agordon/fastx_toolkit.

[40] Bengtsson-Palme J., Ryberg M., Hartmann M., Branco S., Wang Z., Godhe A., De Wit P., Sánchez-García M., Ebersberger I., de Sousa F. (2013). Improved software detection and extraction of ITS1 and ITS2 from ribosomal ITS sequences of fungi and other eukaryotes for analysis of environmental sequencing data. Methods Ecol. Evol..

[41] Rognes T., Flouri T., Nichols B., Quince C., Mahé F. (2016). VSEARCH: A versatile open source tool for metagenomics. PeerJ.

[42] Wang Q., Garrity G.M., Tiedje J.M., Cole J.R. (2007). Naïve Bayesian Classifier for Rapid Assignment of rRNA Sequences into the New Bacterial Taxonomy. Appl. Environ. Microbiol..

[43] Kõljalg U., Nilsson R.H., Abarenkov K., Tedersoo L., Taylor A.F.S., Bahram M., Bates S.T., Bruns T.D., Bengtsson-Palme J., Callaghan T.M. (2013). Towards a unified paradigm for sequence-based identification of fungi. Mol. Ecol..

[44] Nilsson R.H., Tedersoo L., Ryberg M., Kristiansson E., Hartmann M., Unterseher M., Porter T.M., Bengtsson-Palme J., Walker D.M., de Sousa F. (2015). A comprehensive, automatically updated fungal ITS sequence dataset for reference-based chimera control in environmental sequencing efforts. Microbes Environ..

[45] Sha S.P., Suryavanshi M.V., Tamang J.P. (2019). Mycobiome diversity in traditionally prepared starters for alcoholic beverages in India by high-throughput sequencing method. Front. Microbiol..

[46] Nguyen N.H., Song Z., Bates S.T., Branco S., Tedersoo L., Menke J., Schilling J.S., Kennedy P.G. (2016). FUNGuild: An open annotation tool for parsing fungal community datasets by ecological guild. Fungal Ecol..

[47] Henske J.K., Gilmore S.P., Knop D., Cunningham F.J., Sexton J.A., Smallwood C.R., Shutthanandan V., Evans J.E., Theodorou M.K., O’malley M.A. (2017). Transcriptomic characterization of Caecomyces churrovis: A novel, non-rhizoid-forming lignocellulolytic anaerobic fungus. Biotechnol. Biofuels.

[48] Sun W., Yu G., Louie T., Liu T., Zhu C., Xue G., Gao P. (2015). From mesophilic to thermophilic digestion: The transitions of anaerobic bacterial, archaeal, and fungal community structures in sludge and manure samples. Appl. Microbiol. Biotechnol..

[49] Dollhofer V., Callaghan T.M., Griffith G.W., Lebuhn M., Bauer J. (2017). Presence and transcriptional activity of anaerobic fungi in agricultural biogas plants. Bioresour. Technol..

[50] Wilken S.E., Saxena M., Petzold L.R., O’malley M.A. (2018). In silico identification of microbial partners to form consortia with anaerobic fungi. Processes.

[51] Drake H., Ivarsson M. (2018). The role of anaerobic fungi in fundamental biogeochemical cycles in the deep biosphere. Fungal Biol. Rev..

[52] Zhang J., Shi H., Wang Y., Li S., Cao Z., Ji S., He Y., Zhang H. (2017). Effect of dietary forage to concentrate ratios on dynamic profile changes and interactions of ruminal microbiota and metabolites in holstein heifers. Front. Microbiol..

[53] Thongbunrod N., Chaiprasert P. (2024). Potential of enriched and stabilized anaerobic lignocellulolytic fungi coexisting with bacteria and methanogens for enhanced methane production from rice straw. Biomass-Convers. Biorefinery.

[54] Alanbagi R.A., Alshuwaili F.E., Stephenson S.L. (2019). Fungi associated with forest floor litter in northwest Arkansas. Curr. Res. Environ. Appl. Mycol..

[55] Srikanth M., Sandeep T.S.R.S., Sucharitha K., Godi S. (2022). Biodegradation of plastic polymers by fungi: A brief review. Bioresour. Bioprocess..

[56] Taubitz A., Bauer B., Heesemann J., Ebel F. (2007). Role of respiration in the germination process of the pathogenic mold Aspergillus fumigatus. Curr. Microbiol..

[57] Kumar M., Kumar H., Topno R.K., Kumar J. (2019). Analysis of impact of anaerobic condition on the aflatoxin production in Aspergillus parasiticus Speare. Agric. Sci. Dig. A Res. J..

[58] Liu X., Jiang Z., Ma S., Yan Q., Chen Z., Liu H. (2020). High-level production and characterization of a novel β-1,3-1,4-glucanase from Aspergillus awamori and its potential application in the brewing industry. Process. Biochem..

